# Association between Non-High-Density-Lipoprotein-Cholesterol Levels and the Prevalence of Asymptomatic Intracranial Arterial Stenosis

**DOI:** 10.1371/journal.pone.0065229

**Published:** 2013-05-29

**Authors:** Jianwei Wu, Qian Zhang, Huajun Yang, Xiang Gao, Yong Zhou, Anxin Wang, Chunxue Wang, Shufeng Zhang, Shouling Wu, Xingquan Zhao

**Affiliations:** 1 Department of Neurology, Beijing Tiantan Hospital, Capital Medical University, Beijing, China; 2 Department of Neural Stem Cell Transplantation, the General Hospital of Chinese People's Armed Police Forces, Beijing, China; 3 Channing Laboratory, Department of Medicine, Brigham and Women’s Hospital, and Harvard Medical School, Boston, Massachusetts, United States of America; 4 Department of Nutrition, Harvard University School of Public Health, Boston, Massachusetts, United States of America; 5 Department of Neurology, the General Hospital of Chinese People's Armed Police Forces, Beijing, China; 6 Department of Cardiology, Kailuan Hospital, Hebei United University, Tangshan, China; Innsbruck Medical University, Austria

## Abstract

**Objective:**

The aim of this study was to assess the association between non-high-density-lipoprotein-cholesterol (non-HDL-C) and the prevalence of asymptomatic intracranial arterial stenosis (ICAS).

**Methods and Results:**

The Asymptomatic Polyvascular Abnormalities Community (APAC) study is a prospective cohort study based on the Kailuan district (China) population. A total of 5351 eligible subjects, aged ≥40, and without history of stroke or myocardial infarction, were enrolled in this study. Transcranial Doppler Ultrasonography (TCD) was performed on all enrolled subjects for the evaluation of ICAS presence. Out of 5351 patients, 698 subjects showed evidence of ICAS (prevalence of 13.04%). Multivariate analysis showed that non-HDL-C is an independent indicator for the presence of ICAS (OR  = 1.15, 95%CI: 1.08 – 1.23), but with a gender difference (P for interaction<0.01): in men, non-HDL-C is an independent indicator for ICAS (multivariate-adjusted OR  = 1.28, 95%CI: 1.18–1.39), but not in women (multivariate-adjusted OR  = 1.03, 95%CI: 0.93–1.14). Subjects were divided into five subgroups based non-HDL-C levels and these levels correlated linearly with the prevalence of ICAS (P for trend <0.01). Compared with the first quintile, multivariate-adjusted OR (95%CI) of the second, third, fourth and fifth quintiles were: 1.05 (0.71–1.56), 1.33 (0.91–1.95), 1.83 (1.27–2.63), 2.48 (1.72–3.57), respectively.

**Conclusion:**

Non-HDL-C is an independent predictor of ICAS prevalence in men but not in women, suggesting that non-HDL-C levels could be used as a surveillance factor in the primary prevention of ischemic stroke, especially in men.

## Introduction

Intracranial arterial stenosis (ICAS) is an important cause of ischemic stroke and the presence of ICAS is associated with a poor prognosis [1], [2]. In contrast to extracranial atherosclerosis, intracranial atherosclerosis occurs more commonly in Asian [3]. In China, about 30–40% of ischemic strokes and over 50% of transient ischemic attacks (TIA) are associated with the presence of ICAS [1]. Previously identified risk factors for the presence of asymptomatic ICAS include hyperlipidemia, low-density lipoprotein cholesterol (LDL-C) levels, hypertension, smoking and diabetes [4], [5], [6], [7].

Non-high-density lipoprotein cholesterol (non-HDL-C) is a composite marker encompassing all atherogenic apoprotein B-containing lipoproteins, including LDL, very-low-density lipoprotein (VLDL), intermediate-density lipoprotein (IDL), lipoprotein (a), chylomicrons and chylomicrons remnants [8]. Non-HDL-C is an important risk factor for the onset of ischemic stroke [9], [10] and has been shown to be a better risk factor for coronary heart disease than LDL-C [11], [12], [13]. A recent meta-analysis showed that non-HDL-C levels, as a therapeutic target, were more associated to cardiovascular outcomes than LDL-C [14].

However, whether there is an association between non-HDL-C levels and the prevalence of asymptomatic ICAS remains unknown. The purpose of the present epidemiologic and observational study was to evaluate whether non-HDL-C levels can be an indicator of the presence of asymptomatic ICAS in the Chinese adult population.

## Subjects and Methods

### Study Population

The Asymptomatic Polyvascular Abnormalities Community (APAC) study is a community-based, prospective, long-term follow-up observational study, aiming to investigate the epidemiology of asymptomatic polyvascular abnormalities in Chinese adults. A total of 5,852 subjects were randomly sampled from our reference population of 101,510 participants (81,110 males and 20,400 females, aged 18–98 years old) from the Kailuan study[15], which was an ongoing prospective study conducted in 2006 in the Kailuan community owned and managed by the Kailuan Company. The Kailuan Company was founded in 1878, and is located in Tangshan city, a large and littoral modern city located southeast of Beijing. In addition to coal products, the Kailuan Company also manages manufactures, construction activities, electric power, coking, new building materials, chemical industries, bauxite mining, transportation, trading, etc. This study population was selected because of an excellent collaboration from participants and of a good compliance to follow-up visits. Furthermore, because of its variety of activities, the Kailuan community includes individuals from a variety of professional and social classes.

From June 2010 to June 2011, a total of 5816 participants completed the baseline survey. We excluded 376 subjects who could not meet the inclusion criteria: 1) aged 40 years or older; and 2) no history of stroke, transient ischemic attack, and coronary disease at baseline. Finally, a total of 5440 participants were eligible and included in the study. During the baseline survey, all participants underwent extensive clinical examination, laboratory tests and transcranial Doppler (TCD) examinations. We had to exclude 14 participants with incomplete non-HDL-C data and 75 participants under lipid-lowering treatment, leaving 3215 men and 2136 women available for analysis. The study was performed according to the guidelines from the Helsinki Declaration and was approved by the Ethics Committees of the Kailuan General Hospital and the Beijing Tian Tan Hospital. Written informed consent was obtained from all participants.

### Measurement of Indicators

A questionnaire was used to obtain information on enrolled subjects, including age, gender, hypertension, diabetes, smoking, and medications prescribed by physicians. Smoking status was classified into “non-smoking” and “smoking” according to self-reported information.

Hypertension was defined based on: personal history of hypertension, a systolic blood pressure ≥140 mmHg, a diastolic pressure ≥90 mmHg, or currently taking antihypertensive medication prescribed by a physician. Subject height was measured and body mass index (BMI) was calculated as body weight (kg) divided by the squared height (m^2^).

Diabetes mellitus was diagnosed if the subject was undergoing treatment with insulin or oral hypoglycemic agents, if fasting blood glucose (FBG) levels were >126 mg/dl, or if he had a personal history of diabetes mellitus.

### Physical examination

Blood pressure was measured in the sitting position and an average of two readings was used for the current study. If the two measurements differed by more than 5 mm Hg, a third reading was then taken, and the average of the three readings was used.

### Biochemical Index

Blood was drawn after an overnight fast. Plasma samples were prepared and analyzed within 4 h of preparation. Total cholesterol was measured using the endpoint test method [16]. High-density lipoprotein cholesterol (HDL-C) and LDL-C levels were measured using a direct test method [17] and triglycerides (TG) were measured using the GPO method [18] (inter-assay coefficient of variation: <10%; Mind Bioengineering Co. Ltd, Shanghai, China). All blood samples were analyzed using a Hitachi 747 auto-analyzer (Hitachi, Tokyo, Japan) at the Kailuan General Hospital central laboratory. Non-HDL-C levels were determined by subtracting serum HDL-C levels from total cholesterol [19]. Fasting blood glucose (FBG) was measured using the hexokinase/glucose-6-phosphate dehydrogenase method [20].

### Trans-cranial Doppler Ultrasonography (TCD) Examination

**Figure 1:**
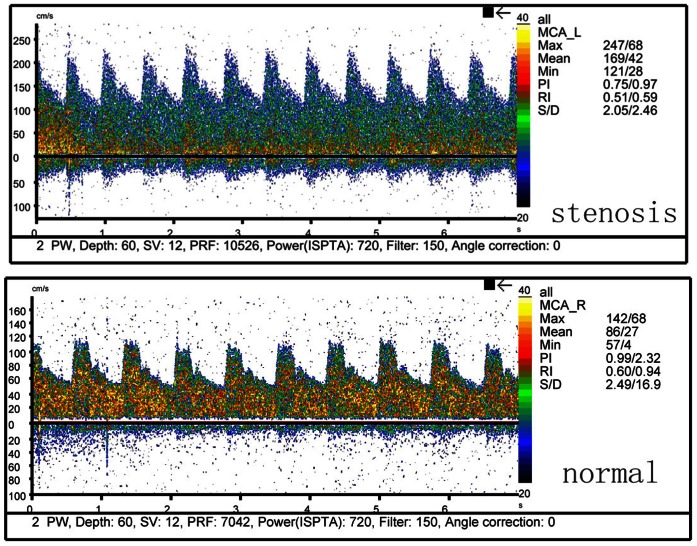
Normal and typical TCD waveform indicative of ICAS in the middle cerebral artery.

TCD was performed by two experienced neurologists using portable devices (EME Companion, Nicolet). ICAS diagnosis was made according to the peak flow velocity criteria that was published and validated against MR angiography and clinical outcomes [21], [22]. Briefly, the occlusive arteries were defined by a peak systolic flow velocity of: >140 cm per second for the middle cerebral artery, >120 cm per second for the anterior cerebral artery, >100 cm per second for the posterior cerebral artery and vertebra-basilar artery, and >120 cm per second for the siphon internal carotid artery. Figure 1 depicts a normal TCD waveform of middle cerebral artery and a typical TCD waveform indicative of ICAS in the middle cerebral artery. In addition to the above criteria, patients’ age, presence of disturbance in echo frequency, turbulence and whether the abnormal velocity was segmental were also taken into consideration for ICAS diagnosis. Subjects without a good temporal window were considered without stenosis. Patients were classified as having occlusive disease if at least one of the studied arteries showed evidence of stenosis or occlusion.

### Statistical analysis

We classified the participants into 5 groups according to serum non-HDL-C quintiles. Continuous variables were compared using analysis of variance (ANOVA) and categorical variables were compared using chi-square tests. The age- and gender-adjusted or multivariate-adjusted odd ratios (ORs) and 95%CI were calculated using logistic regression models. The multivariate-adjusted model was further adjusted for age, gender, BMI, hypertension, diabetes, current smoking status, HDL-C levels and triglycerides. A trend test was used to examine whether there was a dose-dependent relationship between serum non-HDL-C quintiles and ICAS prevalence. Categorical data were treated as continuous data using the median value of non-HDL-C levels in each quintile. Additionally, gender and other potential indicators were also evaluated to assess if there was any significant interaction between these variables and the relationship between non-HDL-C levels and ICAS presence. All statistical analyses were performed using the SAS program package. A P-value <0.05 was considered statistically significant.

## Results

### Prevalence of ICAS

**Figure 2:**
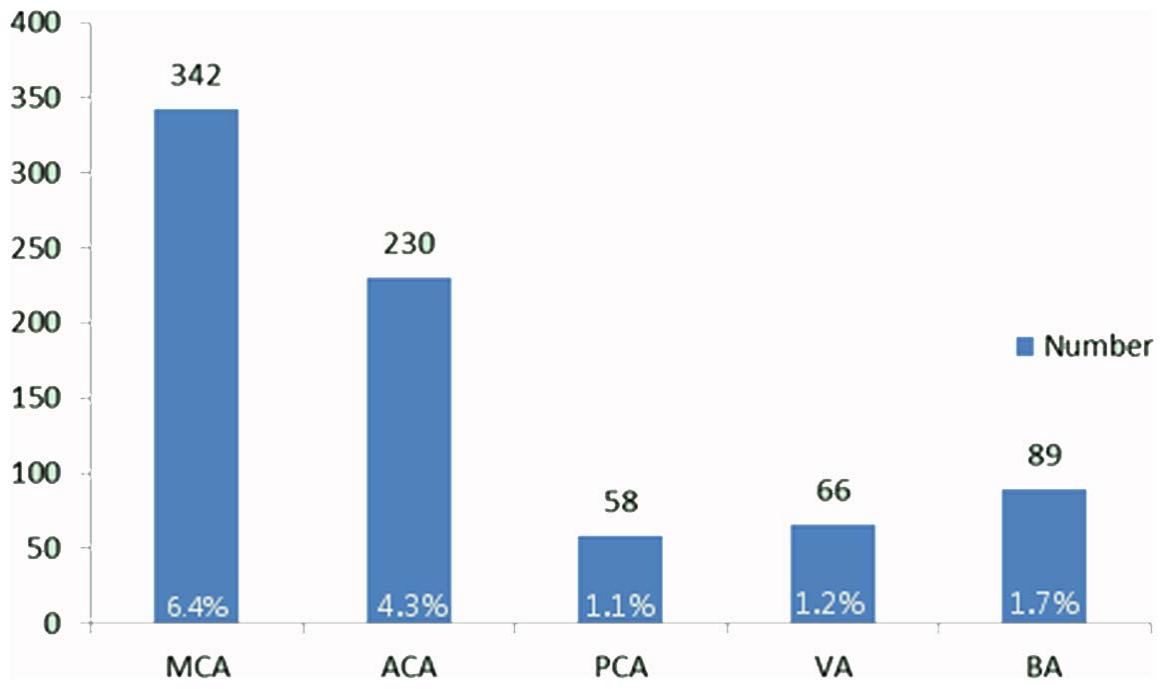
The number (percentages) of stenosis in each vessel segment. MCA: middle cerebral artery, ACA: anterior cerebral artery, PCA: posterior cerebral artery, VA: vertebral artery, BA: basal artery.

Based on TCD results, 698 patients were diagnosed with ICAS, representing a prevalence of 13.0% (698/5351). ICAS patients included 342 with middle cerebral artery stenosis (6.4%), 230 with anterior cerebral artery stenosis (4.3%), 58 with posterior cerebral artery stenosis (1.1%), 66 with vertebral artery stenosis (1.2%), and 89 with basal artery stenosis (1.7%). (Figure 2)

### Baseline characteristics

Subjects’ baseline characteristics are presented in Table 1. The median values of the baseline characteristics are presented for the non-HDL-C levels quintiles subgroups. Non-HDL-C median values were 89.4 mg, 112.3 mg, 128.9 mg, 149.0 mg, and 180.0 mg for quintiles 1 to 5, respectively. There were significant differences for age, gender, BMI, total cholesterol, HDL-C, and triglycerides among quintiles (P<0.01). Absolute values of all these variables, with the exception of HDL-C, continuously increased as non-HDL-C levels increased. In contrast, serum HDL-C levels were lower with higher levels of non-HDL-C. A larger proportion of subjects with higher non-HDL-C levels were men, smokers and had concomitant hypertension and diabetes.

**Table 1:** Median baseline characteristics of participants according to non-HDL-C quintiles.

	non-HDL-C levels, quintiles					P value
	Q1	Q2	Q3	Q4	Q5	
N	1070	1070	1071	1070	1070	
Age, years	50.0	50.7	51.6	53.6	55.0	<0.01
Women, %	45.3	41.1	37.2	36.5	39.4	<0.01
Smoking, %	26.6	31.7	33.1	33.4	35.8	<0.01
Hypertension, %	38.7	44.0	47.6	54.1	54.1	<0.01
Diabetes, %	7.57	9.53	11.2	14.0	17.1	<0.01
Body mass index, kg/m^2^	23.9	24.5	24.7	25.3	25.4	<0.01
Total cholesterol, mg/dl	154.7	173.2	188.7	208.8	242.1	<0.01
HDL-C, mg/dl	66.5	61.1	59.6	58.1	58.1	<0.01
Triglycerides, mg/dl	85.9	101.9	110.8	133.8	164.8	<0.01

Non-HDL-C: non-high-density lipoprotein cholesterol; HDL-C: high-density lipoprotein cholesterol

### Correlation between baseline lipid levels and the prevalence of ICAS

As shown in Table 2, we observed that non-HDL-C is an independent indicator for the presence of asymptomatic ICAS (multivariate-adjusted OR  = 1.15, 95%CI: 1.08 – 1.23). The positive associations between non-HDL-C levels and ICAS diagnosis remained significant after adjustment for age, gender, HDL-C, triglycerides, BMI, smoking, hypertension and diabetes (P for trend <0.01). Compared with quintile 1, prevalence of asymptomatic ICAS was significantly higher in the fourth and fifth quintiles after adjustment for the aforementioned confounding factors (OR = 1.48, 95%CI: 1.12–1.94; OR =  1.67, 95%CI: 1.26–2.20, respectively). In contrast, multivariate analyses showed that the prevalence of ICAS was increased with increasing LDL-C levels (P for trend <0.01). However, compared with the first quintile, the prevalence of asymptomatic ICAS was not significantly higher in the fourth and fifth quintiles (OR = 1.21, 95%CI: 0.94–1.56; OR =  1.29, 95%CI: 0.99–1.66, respectively), suggesting that LDL-C levels may not accurately predict ICAS presence.

**Table 2:** Odd ratios (OR) for ICAS according to baseline blood lipid levels quintiles.

	Median	N (%)	Model1* (95%Cl)	Model2^†^ (95%Cl)
Non-HDL-C				
Q1	89.4	107 (10.0%)	1	1
Q2	112.3	112 (10.5%)	1.08 (0.81–1.43)	1.03 (0.77–1.38)
Q3	128.9	119 (11.1%)	1.11 (0.84–1.47)	1.06 (0.79–1.41)
Q4	149.0	169 (15.8%)	1.60 (1.23–2.08)	1.48 (1.12–1.94)
Q5	180.0	191 (17.9%)	1.74 (1.35–2.26)	1.67 (1.26–2.20)
P for trend			P<0.01	P<0.01
Continuous Scale			1.17 (1.10–1.24)	1.15 (1.08–1.23)
LDL-C				
Q1	66.5	136 (12.9%)	1	1
Q2	87.8	117 (10.7%)	0.87 (0.67–1.14)	0.88 (0.67–1.16)
Q3	100.5	117 (11.1%)	0.96 (0.73–1.26)	0.93 (0.71–1.22)
Q4	113.7	157 (14.6%)	1.26 (0.98–1.62)	1.21 (0.94–1.56)
Q5	136.9	170 (16.1%)	1.35 (1.05–1.73)	1.29 (0.99–1.66)
P for trend			P<0.01	P<0.01
Continuous Scale			1.10 (1.04–1.17)	1.09 (1.03–1.15)

95% CI, 95% confidence interval; non-HDL-C, non-high-density lipoprotein cholesterol; LDL-C, low-density lipoprotein cholesterol.

* Model 1: adjusted for age, and gender. ^†^Model2: adjusted for age, gender, BMI, hypertension, diabetes, smoking, HDL-C and triglycerides.

Further analyses showed that there was a significant difference in the association between non-HDL-C and presence of asymptomatic ICAS between men and women (P for interaction <0.01), and that the association was statistically significant in men only (P for trend <0.01). Our results indicated that in men, the prevalence of ICAS was significantly increased with increasing baseline non-HDL-C levels (OR = 1.28, 95%CI: 1.18–1.39). In contrast, in women, non-HDL-C levels were not an independent indicator for the presence of asymptomatic ICAS (OR = 1.03, 95%CI: 0.93–1.14).When other baseline characteristics (including age, BMI, hypertension, diabetes, and smoking status) were evaluated, our results indicated that the presence or absence of these indicators did not influence the association between non-HDL-C levels and the prevalence of asymptomatic ICAS (P  = 0.08, 0.42, 0.26, 0.07, and 0.11 respectively) (Table 3).

**Table 3:** Multivariate-adjusted odd ratios* (OR) for ICAS according to non-HDL-C levels, stratified by gender and selected risk factors.

	non-HDL-C, quintiles					P for trend	Continuous Scale	P interaction
	Q1	Q2	Q3	Q4	Q5			
Gender								P<0.01
Men	1	1.05 (0.71–1.56)	1.33 (0.91–1.95)	1.83 (1.27–2.63)	2.48 (1.72–3.57)	P<0.01	1.28 (1.18–1.39)	
Women	1	1.06 (0.69–1.62)	0.87 (0.55–1.37)	1.26 (0.82–1.95)	1.04 (0.66–1.64)	P = 0.62	1.03 (0.93–1.14)	
Age								p = 0.08
<60	1	1.10 (0.76–1.58)	0.96 (0.66–1.40)	1.70 (1.20–2.42)	1.44 (0.99–2.10)	P<0.01	1.13 (1.04–1.26)	
≥60	1	0.97 (0.60–1.56)	1.37 (0.87–2.14)	1.39 (0.89–2.16)	2.21 (1.44–3.40)	P<0.01	1.23 (1.11–1.35)	
Hypertension								p = 0.42
No	1	1.30 (0.83–2.05)	1.18 (0.74–1.88)	1.75 (1.11–2.76)	1.55 (0.96–2.50)	P = 0.03	1.12 (1.01–1.25)	
Yes	1	0.88 (0.60–1.28)	0.99 (0.69–1.44)	1.36 (0.96–1.92)	1.71 (1.21–2.42)	P<0.01	1.18 (1.09–1.28)	
Diabetes								p = 0.26
No	1	1.02 (0.75–1.39)	1.01 (0.74–1.37)	1.39 (1.04–1.88)	1.48 (1.09–2.00)	P<0.01	1.12 (1.04–1.20)	
Yes	1	1.20 (0.51–2.81)	1.71 (0.76–3.87)	2.32 (1.08–5.00)	3.45 (1.60–7.42)	P<0.01	1.39 (1.18–1.62)	
Smoking								p = 0.07
No	1	1.18 (0.85–1.64)	0.99 (0.70–1.39)	1.30 (0.94–1.81)	1.50 (1.08–2.09)	P = 0.02	1.10 (1.02–1.18)	
Yes	1	0.71 (0.39–1.29)	1.29 (0.76–2.21)	1.97 (1.18–3.29)	2.14 (1.27–3.59)	P<0.01	1.30 (1.16–1.46)	
BMI, kg/m^2^								p = 0.11
<25	1	0.86 (0.59–1.23)	0.83 (0.59–1.21)	1.20 (0.84–1.71)	1.12 (0.77–1.63)	P = 0.20	1.06 (0.97–1.15)	
≥25	1	1.42 (0.86–2.34)	1.56 (0.96–2.54)	2.11 (1.33–3.35)	2.76 (1.74–4.38)	P<0.01	1.28 (1.16–1.40)	

* Multivariate-adjusted odd ratios (OR): adjusted for age, gender, BMI, hypertension, diabetes, smoking, HDL-C, triglycerides

## Discussion

In this study, we observed that non-HDL-C, but not LDL-C, levels were an independent indicator for the prevalence of asymptomatic ICAS in men, but not in women. To the best of our knowledge, this is the first evidence specifically showing the significant association between non-HDL-C levels and the prevalence of asymptomatic ICAS.

Currently, digital subtraction angiography (DSA), magnetic resonance angiography (MRA) and TCD are methods commonly used to detect and diagnose ICAS. DSA and MRA are not suitable for large epidemiological survey mainly because they are invasive and have to be used in an inconvenient setting. Furthermore, results from a previous study showed that compared with MRA, TCD had a higher sensitivity and specificity for ICAS detection. The sensitivity and specificity of TCD in diagnosing ICAS are 91.4% and 82.7% respectively [23]. TCD is therefore currently considered to be the best method for the continuous monitoring of cerebral hemodynamics as it is non-invasive, simple, and convenient to use with excellent results repeatability [24].

The current study indicated that ICAS prevalence was 13.4% in our study population, which is higher than what was reported from another study conducted in China in 2007 and using a similar study population as well as the same diagnosis criteria for ICAS. The study included 590 asymptomatic villagers aged ≥40 years in rural China and reported an ICAS prevalence of 6.9% [21]. Previous studies assessed the prevalence of symptomatic ICAS in different populations. Tsivgoulis et al. [25] evaluated 467 consecutive patients with ACI (60.4% men, mean age 58 +/– 14 years) in Caucasians, and found that symptomatic intracranial atherosclerosis was documented in 43 patients (9.2%; 95%CI: 6.9%–12.2%). The most common symptomatic intracranial atherosclerosis location was middle cerebral artery (34.9%) followed by terminal internal carotid artery (18.8%). Arenillas et al. [26] reported a symptomatic middle cerebral artery stenosis prevalence of 5.6% in Spain. Meseguer et al. [27] showed that the prevalence of symptomatic ICAS was 8.8% in France. However, it is important to note that these studies examined ICAS in symptomatic populations of patients, while the present study examined asymptomatic ICAS in a general population.

It is possible that the prevalence of ICAS increased in the past five years, which would not be surprising considering the fact that the incidence of dyslipidymia and associated cardiovascular diseases are increasing in modern China [22], [28]. Although BMI, smoking and age were comparable between the present study and the study by Wong et al. [20], there was more hypertensive subjects in the present study (47.7% vs. 22.2%) and slightly more diabetic subjects (11.9% vs. 8.0%). These two differences could explain, at least in part, the increase in asymptomatic ICAS prevalence observed in the present study. Together with our findings that the middle cerebral artery being consistently the most common region of intracranial arterial stenosis, our study provided strong supportive evidence for the importance of identifying indicators associated with the presence of ICAS. However, prospective studies are needed to correctly assess the development of ICAS.

Currently, there are few studies on the relationship between serum lipid levels and ICAS presence. Previous studies in Chinese showed that hyperlipidemia [2] and LDL-C [5] were important risk factors for ICAS. A study conducted in a Korean population also showed that hypercholesterolemia was an independent ICAS risk factor in men [29]. Additionally, serum lipoprotein (a) was also considered as a predictor for intracranial and extra-cranial arterial stenosis in patients with ischemic stroke [30]. However, whether there was an association between non-HDL-C levels and the risk of developing ICAS was not clear. It is worth mentioning that we excluded subjects who were under treatment with lipid-lowering agents; so, measured baseline non-HDL-C levels represent the true levels in our patients.

Non-HDL-C is a mixture of multiple atherogenic apoprotein B-containing lipoproteins, LDL, VLDL, IDL, chylomicrons, chylomicrons remnants, and lipoprotein (a). Previous studies showed that non-HDL-C was an important risk factor for ischemic stroke [9], [10]. Furthermore, the predictive value of non-HDL-C for coronary heart disease is higher than LDL-C [11], [12], [13]. LDL-C is a well- and long-known risk factor for vascular diseases [8], and our study showed that LDL-C levels are indeed associated with the prevalence of ICAS, but that it was significant only for the highest quintile. However, LDL particles are not the only atherogenic apoprotein B-containing lipoproteins in circulation; indeed, studies showed that VLDL, IDL and chylomicron remnants also participate to atherosclerosis [8]. Since LDL-C levels are included in non-HDL-C levels, and since our results showed a better association between non-HDL-C levels and the presence of ICAS, our study provided supportive evidences for this concept as high non-HDL-C levels showed a better association with the presence of asymptomatic ICAS in our study population than LDL-C levels. Recently, the American Diabetes Association and the American Heart Association reached an agreement for lipid management in high-risk patients, in which non-HDL-C is considered better than LDL-C in identifying high-risk patients; it is also recommended that non-HDL-C levels are the primary goal for lipid lowering therapy in high-risk and dyslipidemic patients [31]. From this perspective, our study provided valuable supportive evidence that non-HDL-C should be closely monitored and treated, particularly in men, as it was identified as a clear risk factor for ICAS in this population.

Our findings on gender differences in the association between non-HDL-C levels and the presence of ICAS are also intriguing. The prevalence of ICAS increases with increasing non-HDL-C levels in men, while non-HDL-C was not an indicator of ICAS in women. The differential impact of gender on indicators of ICAS was previously documented. Kim et al. showed that advanced age, diabetes and smoking status were independent risk factors for ICAS in men; in women, only hypertension and diabetes were independent risk factors for the development of ICAS [29]. The potential protecting effects of estrogens on atherosclerosis development may contribute to the observed differential outcome. Previous studies showed that the risk of cardiovascular diseases in premenopausal women was lower than in men of the same age and than in postmenopausal women. This is because estrogens can enhance the effect of carbon monoxide-mediated angiotensin, which has anti-oxidation effect on vascular smooth muscle cells, thus protecting vascular endothelial function and preventing atherosclerosis development [32], [33], [34].

One caveat of the current study is that patients with inadequate temporal window for TCD analysis were considered in the non-ICAS group. This may lead to an underestimation of the ICAS prevalence. Nevertheless, this is the first study investigating the relationship between non-HDL-C levels and the prevalence of ICAS in a large population with a high follow-up ratio and minimal missing information. Additionally, baseline non-HDL-C levels reflect the true lipid levels because subjects under lipid-lowering treatment were excluded. Because of the nature of the Kailuan Company activities, there is an imbalance in gender distribution in the original Kailuan study, men being much more represented than female. We partially overcame this limitation by increasing the women ratio in the APAC study during the random selection process. Also, participants in the Kailuan study received free medical examinations and we cannot exclude a selection bias due to the fact that poorer people were more willing to participate. However, our large sample size should help to generalize our results and our sample was randomly selected from the Kailuan study.

## Conclusion

In conclusion, non-HDL-C levels are an independent indicator of ICAS presence in men. This study provides new evidences supporting the monitoring of non-HDL-C levels as a primary endpoint for stroke prevention. We could obtain the non-HDLC levels by subtracting serum HDL-C levels from total cholesterol; so the measurement of non-HDL-C levels does not lead to cost increase for patients or the healthcare system. We suggest that non-HDL-C evaluation could be used as a screening index as part of the prevention program for stroke and ICAS. However, more epidemiological and experimental data are needed to further confirm this concept.
